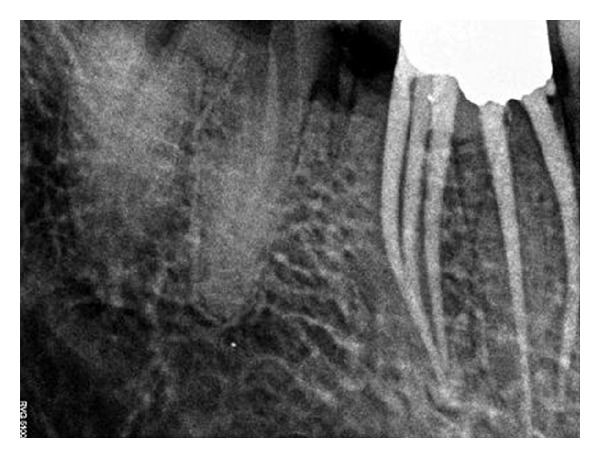# Endodontic Management of Mandibular First Molar with Middle Distal Canal: A Case Report

**DOI:** 10.1155/2012/458079

**Published:** 2012-05-17

**Authors:** Vijay Reddy Venumuddala, M. Sridhar, M. Rajasekaran, Saravanan Poorni, Gnanaprakasam Senthilkumaran

**Affiliations:** ^1^Department of Conservative Dentistry and Endodontics, Hi-Tech Dental College and Hospital, Bhubneshwar 600119, India; ^2^Department of Conservative Dentistry and Endodontics, Ragas Dental College, Chennai 602024, India; ^3^Department of Conservative Dentistry and Endodontics, Sri Venkateshwara Dental College and Hospitals, Chennai 603103, India

## Abstract

The knowledge of variations in root canal morphology is critical for a successful endodontic treatment. This article presents the endodontic management of a unique case of mandibular molar with middle distal canal which is quite uncommon.

## 1. Introduction

Consistently high levels of success in endodontic treatment require an understanding of the root canal anatomy and morphology and that the entire root canal system must be debrided, disinfected, and filled. Thus, it is necessary for the clinician to have knowledge of not only the normal anatomy but also its variations [1]. 

Anatomical characteristics of permanent mandibular molars are generally described as a group of teeth with two roots. The usual canal distribution is two canals in the mesial root and one or two in the distal root [2]. The variations in the normal anatomy of mandibular molar have been extensively studied in the literature [2–7]. The incidence of five canals in the mandibular first molar has been reported to vary between 1% and 15% whereas the incidence of three distal canals has been reported to be much lower at 0.6% [8, 9]. Although Stroner et al. [3] have reported the presence of three distal canals in mandibular first molar as early as 1984, yet literature reveals paucity in the reports on the occurrence of middle distal canal in mandibular molars. This case report describes the diagnosis and successful management of a case of mandibular first molar with this unusual morphological variation of three distal canals.

## 2. Case Report

A 40-year-old male patient reported to the Department of Conservative Dentistry and Endodontics, HiTech Dental college and Hospitals, complaint of pain in the posterior right mandibular region for the past two weeks. He gave a history of intermittent pain in the same region for the past three months since the tooth was restored. His past medical history was found to be noncontributory. Clinical examination revealed a carious right mandibular first molar (47) with tenderness on percussion. The clinical findings, radiographic findings, and vitality tests led to a diagnosis of irreversible pulpitis with acute apical periodontitis of the right mandibular first molar, necessitating endodontic therapy.

**Figure 1:**
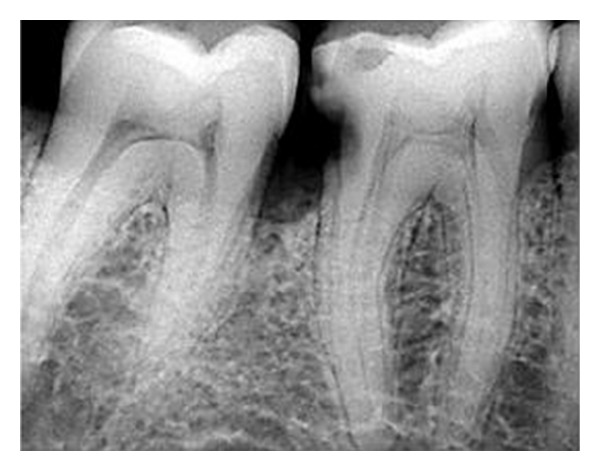


**Figure 2:**
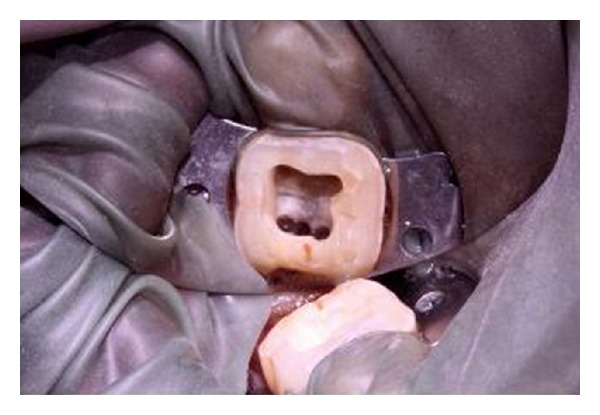


**Figure 3:**
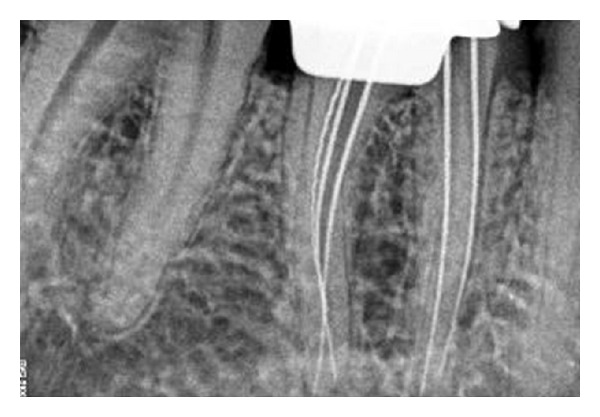


Radiographic evaluation of the involved that tooth did not reveal any unusual anatomy (Figure 1). The tooth was anesthetized using 2% Lignocaine with 1 : 80,000 adrenaline (Lignox, Indoco Remedies Ltd., India) and isolated using rubber dam. Endodontic access cavity was established. The pulp chamber frequently flushed with 5% sodium hypochlorite to remove debris and bacteria. Inspection of the pulp chamber revealed five canal orifices (2 mesial and 3 distal, Figure 2). Canal patency was checked with number 10 K-file (Mani, Inc.; Tochigi, Japan). Working length radiograph was taken (Figure 3) and the presence of five canals was confirmed.

**Figure 4:**
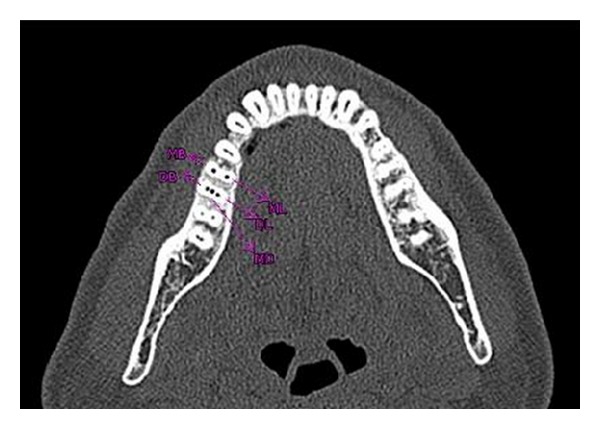


**Figure 5:**
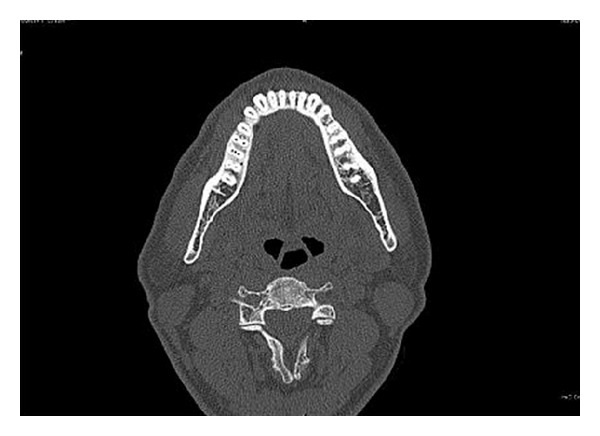


Cleaning and shaping was performed using using a crown down technique with Protaper files (Maillefer, Dentsply, Ballaigues, Switzerland) under abundant irrigation with 5% sodium hypochlorite solution in a 5 mL syringe and EDTA (Glyde, Maillefer, Dentsply, Ballaigues, Switzerland). The tooth was then temporized. In order to ascertain the presence of middle distal canal, dental imaging using a multisliced computed tomography (SCT) was planned. After obtaining the informed consent from the patient, SCT of the mandible was performed using Dentascan, Dental Software (GE healthcare, USA). The spiral computed tomographic images revealed the presence of the three distal canals (Figures 4 and 5).

Patient was then recalled after a week. The root canals were then dried with paper points (Maillefer, Dentsply, Ballaigues, Switzerland) and obturated with cold, laterally condensed gutta-percha (Maillefer, Dentsply, Ballaigues, Switzerland) and AH plus resin sealer (Maillefer, Dentsply, Ballaigues, Switzerland, Figure 6).

## 3. Discussion

The main objective of root canal treatment is the thorough mechanical and chemical cleansing of the entire pulp cavity and its complete obturation with an inert filling material and a coronal filling preventing ingress of microorganisms. According to Ingle, one of the most important causes of endodontic treatment failure is the incomplete obturation of the root canal system [10]. Similarly, Vertucci reported that a considerable number of failures could be assigned to anatomical variations, such as the presence of canals not usually found [11]. Therefore, the correct location, thorough debridement, cleaning, shaping, and obturating the entire root canal system are indispensable procedures.

It has been postulated that secondary dentin apposition during tooth maturation would form dentinal vertical partitions inside the root canal cavity, thus creating root canals. A third root canal may also be created inside the root canal cavity of mandibular molars by this process. Such third canals are situated centrally between the two main buccal and lingual root canals. The diameter of those third middle canals is smaller than that of the other two [12]. The incidence of third canal in the distal root of mandibular molars was found to be much lower than in the mesial root as reported byMartínez-Bernáand Badanelli [12]. The larger mesiodistal dimension of the distal root, compared to mesial root, may account for the rare incidence of third canal created by dentine apposition in distal roots. 

Variations in the distal root of mandibular first molars can be identified through very careful observation of angled radiographs. Buccolingual views, 20° from mesial and 20° from distal reveal the basic information on the tooth's anatomy and root canal system required for endodontic treatment [13]. Interpretation and appraisal based on a 2D radiograph may alert the clinician to the presence of aberrant anatomy but would not be able to present the variable morphologic structure of root canals and their interrelations. [14]

Three dimensional models, which make possible observations from arbitrary viewpoints, are replacing two-dimensional methods for the morphological study of pulp space. Nance et al. reported that tuned aperture CT imaging enabled a statistically significant increase in canal detection as compared with the conventional radiography[15].Spiral computed tomography (SCT) is a recent advancement in the CT technology wherein a 3D data set is acquired and then reconstructed into images representing transverse section of the object. Images can be easily reconstructed into different planes if thin sections are obtained initially [16]. In the present case, we have used noninvasive higher end diagnostic aid, SCT to evaluate the third canal in the distal root of mandibular second molar due to its technical advantages.

## 4. Conclusion

Endodontic success in teeth with the number of canals above that normally found requires a correct diagnosis and careful inspection.  Morphological variations in pulpal anatomy must always be considered before beginning treatment. The case presented shows that a middle distal canal in mandibular molars is one such variations. Although the frequency is rare, each case should be evaluated carefully both clinically and radiographically.

## Figures and Tables

**Figure 6:**